# Thermal Conductivity of Two Types of 2D Carbon Allotropes: a Molecular Dynamics Study

**DOI:** 10.1186/s11671-018-2831-8

**Published:** 2019-01-07

**Authors:** Shanchen Li, Hongru Ren, Yue Zhang, Xiangwei Xie, Kun Cai, Chun Li, Ning Wei

**Affiliations:** 1 Key Laboratory of Agricultural Soil and Water Engineering in Arid and Semiarid Areas, Ministry of Education, Northwest A&F University, Yangling, 712100 China; 2 School of Mechanics, Civil Engineering and Architecture, Northwestern Polytechnical University, Xi’an, 710072 China; 3 Centre for Innovative Structures and Materials, School of Engineering, RMIT University, Melbourne, 3800 Australia

**Keywords:** Two-dimensional material, Molecular dynamics, Thermal conductivity, Carbon allotrope

## Abstract

The thermal properties of the two novel 2D carbon allotropes with five-five-eight-membered rings are explored using molecular dynamics simulations. Our results reveal that the thermal conductivity increases monotonically with increasing size. The thermal conductivities of infinite sizes are obtained by linear relationships of the inverse length and inverse thermal conductivity. The converged thermal conductivity obtained by extrapolation in the reverse non-equilibrium molecular dynamics method is found to be in reasonable agreement with that in the equilibrium molecular dynamics method. The much lower thermal conductivity, compared with graphene, is attributed to the lower phonon group velocity and phonon mean free path. Temperature and strain effects on thermal conductivity are also explored. The thermal conductivity decreases with increasing temperature and it can also be tuned through strain engineering in a large range. The effect of strain on TC is well explained by spectra analysis of phonon vibration. This study provides physical insight into thermal properties of the two carbon allotropes under different conditions and offers design guidelines for applications of novel two-dimensional carbon allotropes related devices.

## Introduction

The carbon materials, e.g., diamond [1], carbon nanotubes [2–5], and graphene [6–12], have stimulated tremendous research interests due to their excellent thermal transport properties. Especially the low-dimensional carbon materials show outstanding properties in heat transport. As a 1D material, the high thermal conductivity (TC) of a single carbon nanotube has been observed by experiments [2, 3], and theoretical studies [4, 5]. Moreover, as a single-atom-thick flat two-dimensional (2D) carbon material, graphene is considered as a revolutionary material for the future generation of thermal conductive reinforced composites due to its high TC [6–12]. It is also reported that the TC of graphyne can reach 40% of graphene and it has potential applications in thermal management [13–15].

Inspired by the fascinating characteristics of these carbon allotropes, researchers have made intensive efforts to study the carbon allotropes and their derivatives in recent years. The experimental and theoretical approaches have been adopted to investigate the novel 2D carbon allotropes, such as the sp^2^-like carbon layer with five-, six-, and seven-membered rings [16]; 2D amorphous carbon with four-membered rings [17]; planar carbon pentaheptite [18]; 2D carbon semiconductor with patterned defects [19]; several 2D flat carbon networks [20]; octagraphene [21]; T-graphene [22]; and H-net [23]. Identifications of the unique properties of these 2D carbon allotropes are significant for future generations of nanomaterials in electronic, photonic, and thermal fields [16–23].

With growing interest in exploring new structures of the 2D carbon allotropes, Su et al. [24] proposed two novel energetically competitive and kinetically stable 2D carbon allotropes composed of octagons and pentagons via the first-principle calculation. The kinetic stability of these two carbon sheets was confirmed by calculating their phonon dispersion curves. Due to the fact that structures of these two carbon allotropes can be viewed as copying the five-five-eight-membered rings (558) ribbon along a straight line path and along a zigzag path, these two carbon allotropes are thus named as octagon and pentagon graphene-line (OPG-L) and octagon and pentagon graphene-zigzag (OPG-Z), respectively. The formation energy of these two carbon allotropes are 0.31 eV/atom and 0.34 eV/atom, respectively. The values are much lower than the formation energy of previously synthesized graphyne, i.e., 0.76 eV/atom [25]. It is noted that the OPG-Z possesses remarkable anisotropy of electronic structure which has potential applications in electronic devices [24]. Consequently, to meet the requirements of electronic applications of OPG-L and OPG-Z, it is inevitable and necessary to research the thermal dissipation properties of the two novel structures. Till now, the thermal properties of these two structures are still not clear.

In this work, we investigate the thermal properties of the two novel 2D carbon allotropes using molecular dynamics simulations. Size, strain, and temperature effects on TC are explored. The results are analyzed by calculating the vibration density of states (VDOS) of phonons. Our research of the thermal properties of these two carbon allotropes indicates their potential applications in thermal management devices.

## Model and Methods

**Fig. 1:**
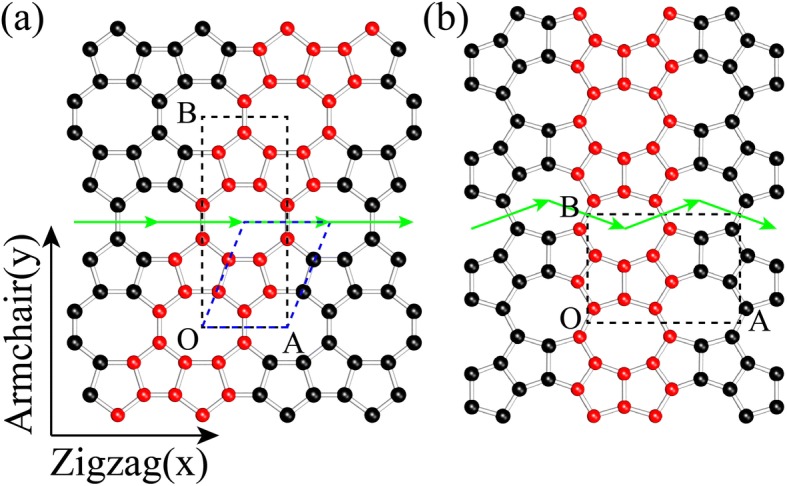
The schematic models of **a** OPG-L and **b** OPG-Z. The black dashed frames are the orthogonal unit cells of OPG-L and OPG-Z, where OA and OB are lattice vectors. The primitive cell of OPG-L is shown in blue dashed frame while the primitive cell of OPG-Z is the same as the crystal cell

The structures of OPG-L (Fig. 1a) and OPG-Z (Fig. 1b) contain representative cells composed of octagons and pentagons [24]. In order to distinguish the edge types of the structures, we define the chirality of armchair and zigzag just like graphene (see Fig. 1). These two structures can be formed by the representative 558 ribbon indicated by the red atoms using translational symmetry along the green rows.

All MD simulations are performed using the large-scale atomic/molecular massively parallel simulator (LAMMPS) package [26]. We use the optimized Tersoff potential by Lindsay and Broido [27], with small modifications, i.e., modified optimized Tersoff potential, to describe the interactions among the carbon atoms. Lindsay and Brodio optimized two parameters compared to the original Tersoff potential [28], one for the equilibrium bond angle and one for the attractive interaction strength. According to this optimized Tersoff potential [27], the equilibrium bond length in graphene is 1.4388 Å, which is larger than the experimental value of 1.42 Å [29]. Because the only length-related parameters in the Tersoff potential are *λ*_1_ in the repulsive function (*f*^*R*^ = *A* exp.(-*λ*_1_*r*)) and *λ*_2_ in the attractive function (*f*^*A*^ = *B* exp(-*λ*_2_*r*)), we can obtain the correct bond length by multiplying these two parameters by a factor of 1.4388/1.42. That is, we change *λ*_1_ from 3.4879 Å^−1^ to 3.5333 Å^−1^ and change *λ*_2_ from 2.2119 Å^−1^ to 2.2407 Å^− 1^. These modifications only change the length scale of the potential in a global way. Based on this modified optimized Tersoff potential, the corresponding equilibrium lattice parameters in MD simulation are as follows: OA = 3.63 Å, OB = 9.38 Å in OPG-L and OA = 6.78 Å, OB = 5.04 Å in OPG-Z, which are in good agreement with the previous study of Su et al. [24], i.e., OA = 3.68 Å, OB = 9.12 Å in OPG-L and OA = 6.90 Å, OB = 4.87 Å in OPG-Z.

**Fig. 2:**
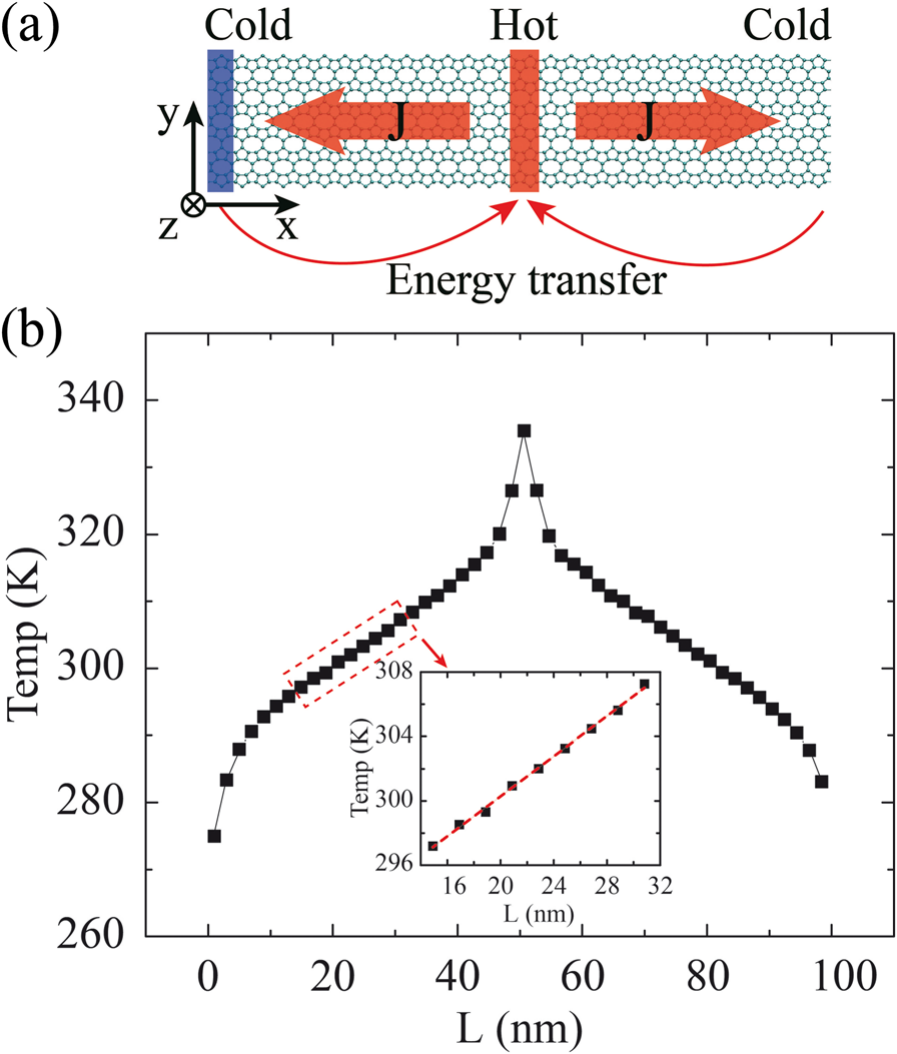
**a** Schematic plot of the rNEMD method. The heat flux transfers from the heat source (hot region) to the heat sink (cold region). The heat flux transport direction is defined as the length direction (L) while the transverse direction is the width (W) direction. **b** The distribution of average temperature as a function of the slabs

Reverse non-equilibrium molecular dynamics (rNEMD) [30] simulations are performed to calculate the TC. The periodic boundary conditions are adopted in x and y dimensions. The structures of OPG-L and OPG-Z are initially optimized via the Polak-Ribiered version of conjugated gradient algorithm [31], and a 0.25-ns Nosé-Hoover thermal bath [32, 33] is employed later to ensure the system reaches the equilibrium state at 300 K (with a time step of 0.25 fs). After approaching the equilibrium state, the model is divided into 50 slabs along the heat transfer direction. As shown in Fig. 2a, the 1st slab is assigned to be the heat sink while the 26th (middle slab of the sample) is the heat source, and the heat flux transfers from the heat source (hot region) to the heat sink (cold region). The heat flux transport direction is defined as the length direction (L) while the transverse direction is the width (W) direction. The heat flux J is released/injected between these two slabs by exchanging the kinetic energies between the hottest atom, which has the highest kinetic energy, in the heat sink slab and the coldest atom, which has the lowest kinetic energy, in the heat source slab. The heat flux J can be obtained by calculating the exchanging amount of the kinetic energy between the heat sink and the heat source slab according to the following equations.

1 $$ J\kern0.5em =\kern0.5em \frac{\sum_{\mathrm{Nswap}}\frac{1}{2}\left({mv}_h^2-{mv}_c^2\right)}{t_{\mathrm{swap}}}, $$

where *t*_swap_ is the total time of exchanging kinetic energy, *N*_swap_ denotes the amount of exchanging atoms pairs, *m* is the mass of atom, and *v*_*h*_ and *v*_*c*_ represent the velocity of exchanging atoms (the hottest atom with the highest kinetic energy in the heat sink slab and the coldest atom with the lowest kinetic energy in the heat source slab), respectively. The temperature of each slab is collected and averaged over 3.0 ns to obtain temperature distribution when system reaches non-equilibrium steady state (after 1.5 ns). The value of TC (*κ*) is then calculated by using the Fourier’s law as

2 $$ \kappa =\frac{J}{2A\partial T/\partial L}, $$

where *A* is the cross-sectional area of heat transfer (*A* is obtained by multiplying the width and thickness of the model), and *∂T*/*∂L* denotes the temperature gradient after the system reaches non-equilibrium steady state (see Fig. 2b). The factor 2 represents the fact that the heat flux transports in two directions away from the heat source. The thickness of model is assumed to be the interlayer equilibrium spacing of graphene (0.34 nm) [8, 10, 34, 35].

## Results and Discussions

**Fig. 3:**
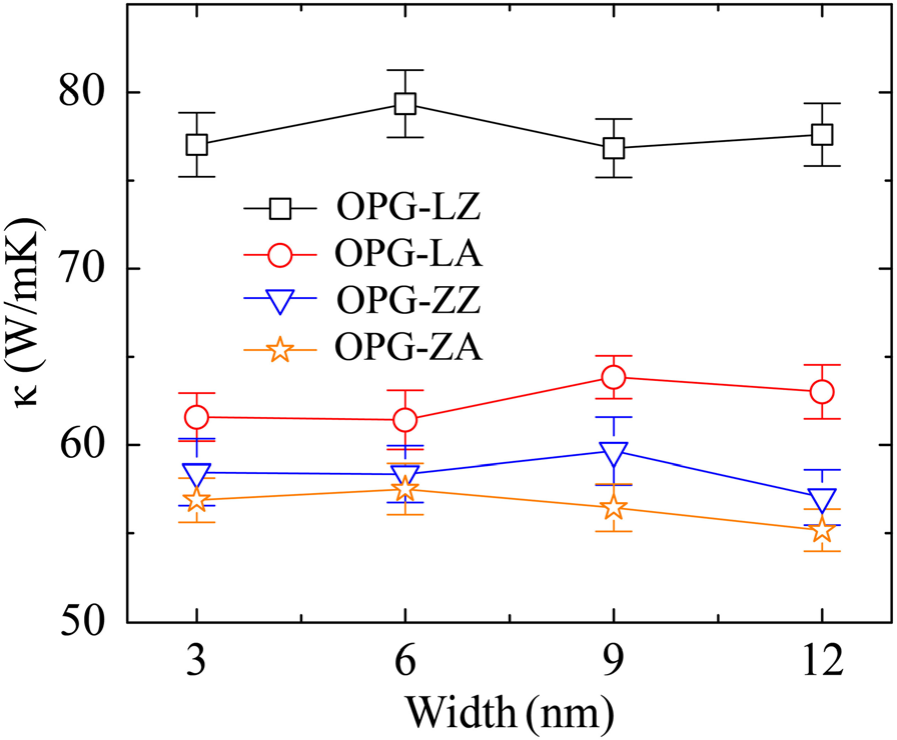
TC of OPG-L and OPG-Z as a function of width

We first examine the system size effect on the TC of the two carbon allotropes. Simulation samples are generated with the same width of 3 nm but different length varying from 50 to 1000 nm. It should be noted that all of the values of the sample length mentioned in this work are the effective length (*L*_*eff*_) of heat transfer. That is, the effective sample length is half of the sample length (*L*), i.e., *L*_*eff*_ = *L*/2, which is attributed to the heat flux transferring from the middle (the heat source) to the both ends (the heat sink) of the sample in the rNEMD method. Particularly, we have confirmed that the TC does not depend on the sample width by calculating the thermal conductivities of samples with fixed length of 50 nm but different width of 3 nm, 6 nm, 9 nm, and 12 nm, respectively, as shown in Fig. 3. The TC of OPG-L along the zigzag and the armchair directions are named as *κ*_OPG-LZ_ and *κ*_OPG-LA_, respectively. Similarly, *κ*_OPG-ZZ_ and *κ*_OPG-ZA_ are used to represent the TC of OPG-Z along the zigzag and the armchair directions. The simulation results show that the TC of OPG-L and OPG-Z in the two chiral directions increases monotonically with sample length varying from 50 to 1000 nm. It is attributed to that in the long sample, the acoustic phonons with longer wave-length are involved to heat transfer [9, 36]. Respectively, the TC of 50-nm- and 1000-nm-long OPG-L and OPG-Z along the zigzag direction are *κ*_OPG-LZ50_ = 125 W/mK, *κ*_OPG-LZ1000_ = 296 W/mK, *κ*_OPG-ZZ50_ = 94 W/mK, and *κ*_OPG-ZZ1000_ = 236 W/mK. Along the armchair direction, the TC of OPG-L and OPG-Z are *κ*_OPG-LA50_ = 105 W/mK, *κ*_OPG-LA1000_ = 316 W/mK, *κ*_OPG-ZA50_ = 93 W/mK, and *κ*_OPG-ZA1000_ = 214 W/mK.

**Fig. 4:**
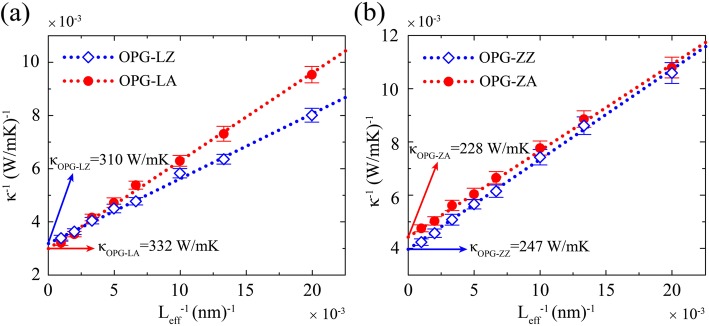
Inverse TC of **a** OPG-L and **b** OPG-Z as a function of the inverse length of the sample at 300 K. The open blue diamond and red spots represent TC along the zigzag and the armchair directions, respectively

In order to extract the TC of infinitely long samples, an inverse fitting procedure is employed. The relationship between the inverse length and inverse TC is expressed as [37–39]:

3 $$ {\kappa}^{-1}=\kappa {}_{\infty }{}^{-1}\left(\frac{2l}{L_{eff}}+1\right), $$

where *κ*_∞_ is the extrapolated TC of an infinite sample, *l* is the phonon mean free path, and *L*_*eff*_ is the effective length of heat transfer. Equation (3) suggests that the relationship between the inverse length and inverse TC should be linear. As shown in Fig. 4, a linear relationship between the inverse length and inverse TC is observed. By extrapolating to L^−1^ = 0, the TC of infinite samples, i.e., *κ*_OPG-LZ_ = 310 W/mK, *κ*_OPG-LA_ = 332 W/mK, *κ*_OPG-ZZ_ = 247 W/mK, and *κ*_OPG-ZA_ = 228 W/mK, are obtained.

**Fig. 5:**
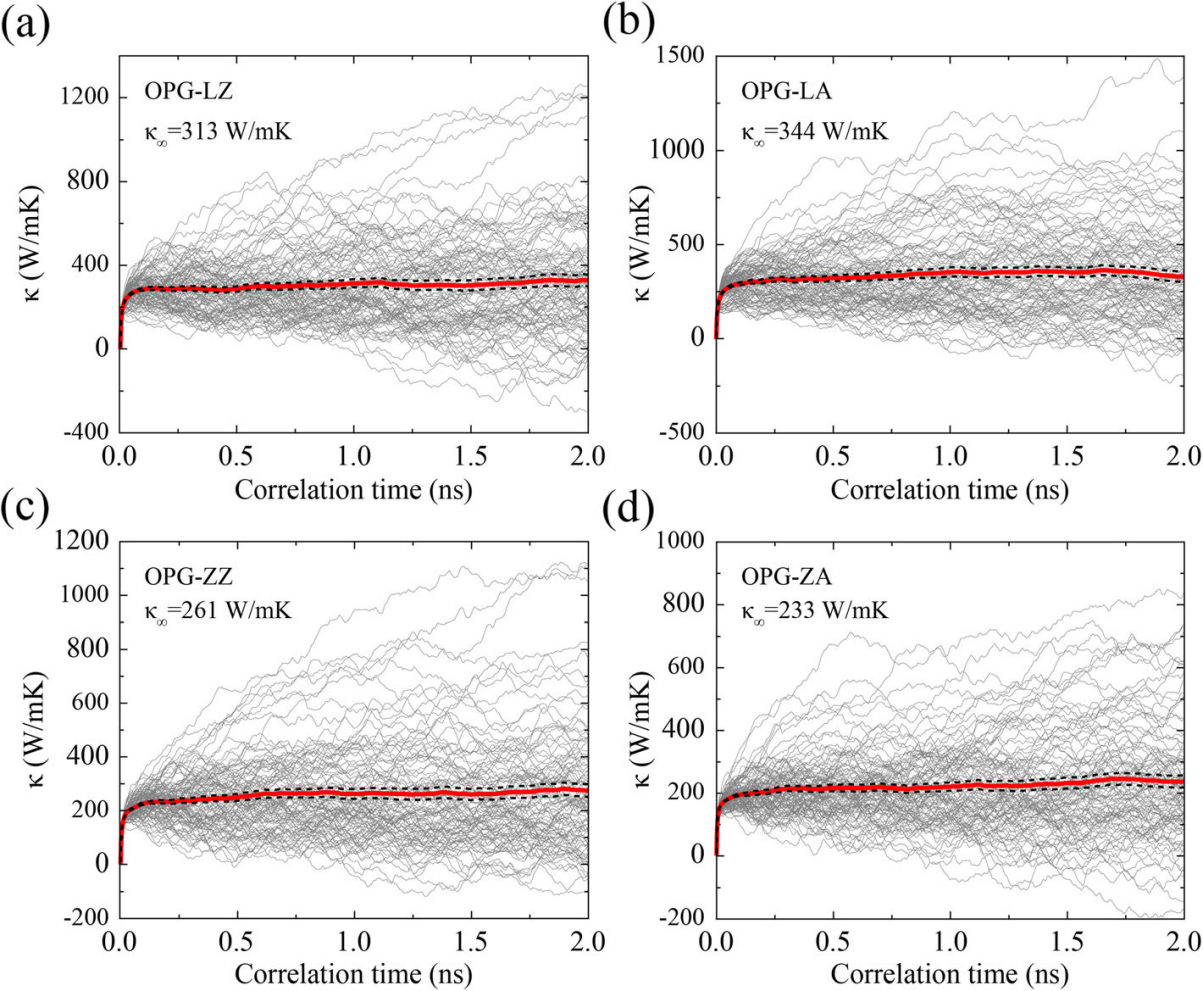
Evolution of TC of **a** OPG-LZ, **b** OPG-LA, **c** OPG-ZZ, and **d** OPG-ZA at 300 K as a function of correlation time. The thin lines represent the results from 100 independent simulations and thick solid and dashed lines represent their average and error bounds. *κ*_∞_ is the TC of an infinite sample, which is obtained by averaging the running TC in correlation time from 1.0 to 2.0 ns

In addition, we also express the running TC in the equilibrium molecular dynamics (EMD) method by establishing the sample with the same length and width of 20 nm (this simulation sample size has been tested to be large enough to eliminate finite-size effects). According to the work by Fan et al. [39, 40], the TC calculations in the EMD method is based on the Green-Kubo formula [41, 42], in which the running TC along the x direction can be expressed as follows:

4 $$ {\kappa}_{xx}(t)=\frac{1}{\kappa_B{T}^2V}{\int}_0^t\left\langle {J}_x(0){J}_x\left({t}^{\hbox{'}}\right)\right\rangle {dt}^{\hbox{'}}, $$

where *κ*_*B*_ is the Boltzmann’s constant, *V* is the volume of the system, *T* is the absolute temperature of the system, 〈*J*_*x*_(0)*J*_*x*_(*t*^'^)〉 is the heat flux autocorrelation function, *t* is the correlation time, and *J*_*x*_ is the heat flux in the x direction. The symbol 〈〉 represents the time average in EMD simulations. The maximum correlation time is 2 ns, which has been tested to be large enough. As shown in Fig. 5, the running TC for OPG-L and OPG-Z at two chiral directions at 300 K are expressed by averaging the results of 100 independent simulations with different initial velocity. We can further obtain the TC of an infinite sample by averaging the running TC in correlation time from 1.0 to 2.0 ns. That is, the converged TC of OPG-LZ, OPG-LA, OPG-ZZ, and OPG-ZA are 313 W/mK, 344 W/mK, 261 W/mK, and 233 W/mK, respectively, which are in reasonable agreement with the results by extrapolation in the rNEMD method.

It is found that the TC of these two carbon allotropes is much lower than that of graphene (3000–5000 W/mK) [7, 43]**.** To explain this phenomenon and explore physical insight, we calculate three important parameters, i.e., *C*_*v*_, *v*_*g*_, and *l*, based on the classical lattice thermal transport equation:

5 $$ \kappa =\frac{1}{3}{C}_v{v}_gl, $$

where C_v_ is heat capacity, v_g_ is effective phonon group velocity, and l is phonon mean free path.

**Fig. 6:**
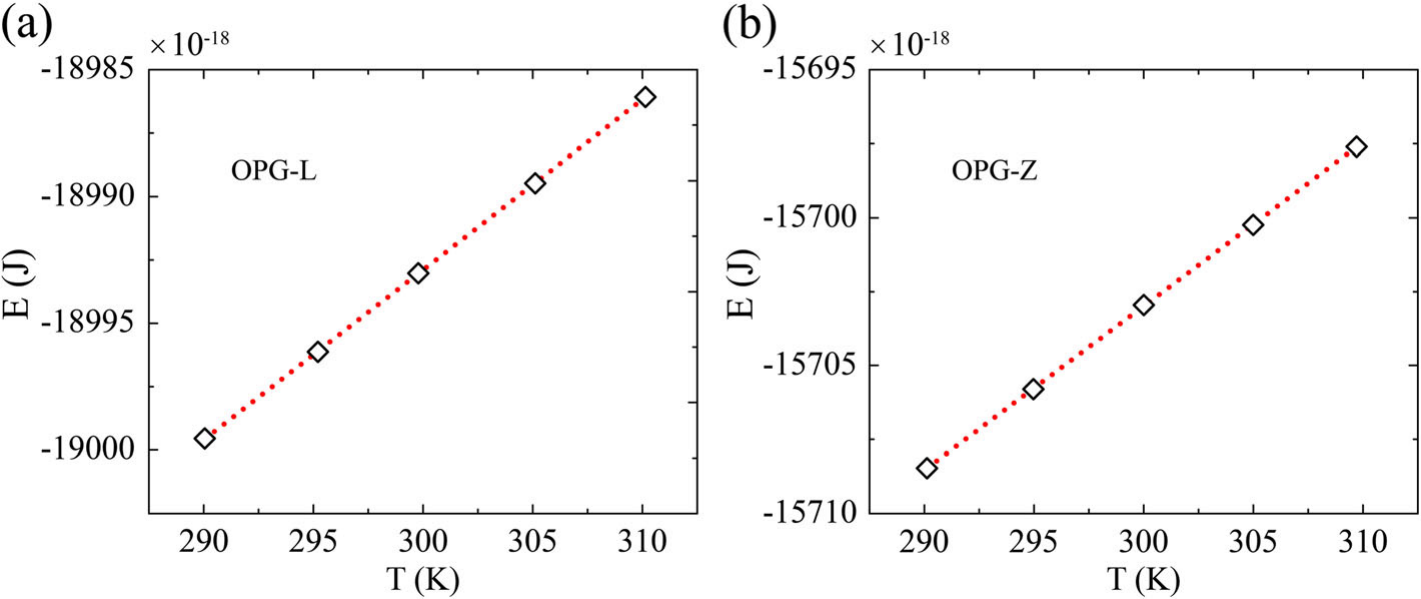
The variation of energy as a function of temperature for **a** OPG-L and **b** OPG-Z. The slope of the energy-temperature curve denotes the heat capacity. The corresponding heat capacities are 4.163 E-23 J/K, and 4.126 E-23 J/K, respectively, per atom

The sample with both length and width of 20 nm is adopted to investigate the heat capacity at 300 K. The heat capacity is computed following the approach of McGaughey and Kaviany [44], which has been used in the approach-to-equilibrium molecular dynamics simulations [45]. We calculate the total energy *E* at temperature of *T* = 290 K, 295 K, 300 K, 305 K, 310 K in the canonical ensemble, and the results are averaged over 60 ps of ten independent simulations with different initial velocity. As shown in Fig. 6, the slope in the linear fitting of energy-temperature curve is the heat capacity.

**Fig. 7:**
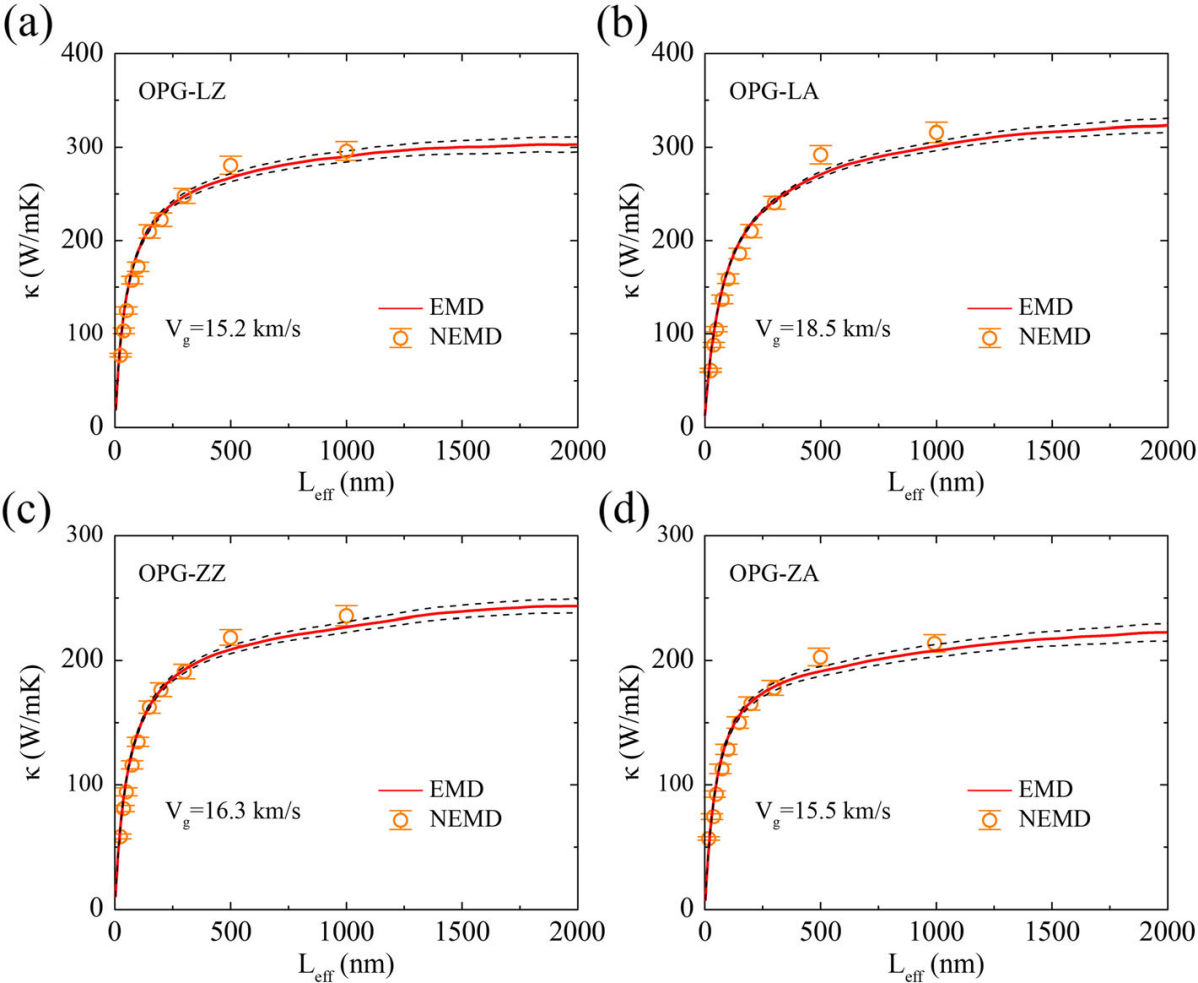
TC of **a** OPG-LZ, **b** OPG-LA, **c** OPG-ZZ, and (**d**) OPG-ZA as a function of effective sample length from EMD and rNEMD simulations. The effective phonon group velocity *V*_*g*_ is obtained by combining EMD and rNEMD simulations

It should be noted that the phonon group velocity we calculate here is the effective phonon group velocity *v*_*g*_ rather than average phonon group velocity *v*. As shown in Fig. 7, the effective phonon group velocity can be obtained by comparing the results of the rNEMD and the EMD simulations. That is, an effective system length *L*_*eff*_ can be defined in the EMD method by multiplying the upper limit of the correlation time *t* in the Green-Kubo formula Eq. (4) by an effective phonon group velocity *v*_*g*_, *L*_*eff*_ ≈ *v*_*g*_*t*. The running TC *κ*(*t*) of the EMD method can also be regarded as a function of the system length *κ*(*L*_*eff*_). In comparison with the average phonon group velocity, the effective phonon group velocity is rough estimate, but it has been extensively used in studying thermal transport in low-dimensional lattice models [46] and has also been used for graphene [40] and allotropes of Si [39].

Based on Eq. (3), the phonon mean free path can be obtained by extrapolation in the rNEMD method. To compare the TC of these two carbon allotropes with that of graphene, we also present these three parameters of graphene. The heat capacity of graphene is calculated through the above method while the effective phonon group velocity and phonon mean free path are obtained in other works [7, 40]. It can be found that the heat capacities of these two carbon allotropes are close to that of graphene; however, the effective phonon group velocity and phonon mean free path are much lower than that of graphene, which leads to the lower TC of the two materials (see Table 1).

**Table 1:** Three parameters, heat capacity *C*_*v*_, effective phonon group velocity *v*_*g*_, and phonon mean free path *l*, of these two carbon allotropes and graphene. *N* is the total number of atoms in the sample

Properties	OPG-LZ	OPG-LA	OPG-ZZ	OPG-ZA	Graphene	Unit
*C*_*v*_	4.163	4.163	4.126	4.126	4.172	10^−23^ J/KN
*v*_*g*_	15.2	18.5	16.3	15.5	57 [40]	km/s
*l*	37.6	55.0	41.6	37.0	775 [7]	nm

**Fig. 8:**
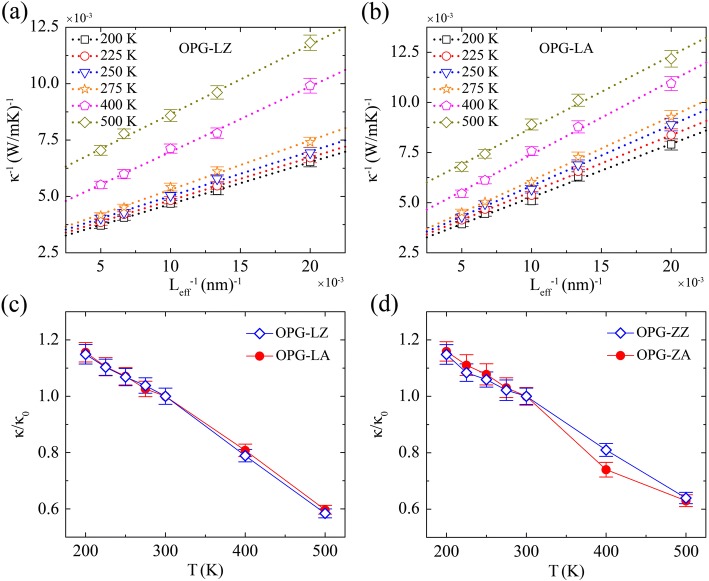
Inverse TC of **a** OPG-LZ, **b** OPG-LA at various temperatures as a function of the inverse sample length, and the relative TC (*κ*/*κ*_0_) of **c** OPG-L and **d** OPG-Z as a function of temperature. *κ*_0_ is the TC at 300 K, which is 310 W/mK, 332 W/mK, 247 W/mK, and 227 W/mK for *κ*_OPG-LZ_, *κ*_OPG-LA_, *κ*_OPG-ZZ_, and *κ*_OPG-ZA_, respectively

Furthermore, we explore the dependence of TC on the temperature, as shown in Fig. 8. The temperature region of 200 K to 300 K is the major range that we focus on. Simulation samples are generated with the same width of 3 nm but different length of 50 nm, 75 nm, 100 nm, 150 nm, and 200 nm, respectively. As shown in Fig. 8a, b, we give the inverse TC of OPG-LZ and OPG-LA at various temperatures as a function of the inverse sample length. Similar to the extrapolation in size effect at 300 K, the thermal conductivities of an infinite sample at various temperatures are extracted by doing extrapolation procedure. As shown in Fig. 8c, d, all the converged thermal conductivities are normalized by the TC at 300 K (*κ*_0_).

Figure 8 indicates that along both the zigzag and the armchair directions, the TC decreases with increasing temperature for both OPG-L and OPG-Z. The trend of TC varies with temperature (from 200 to 500 K) is in good agreement with those of previous TC studies of graphene [8, 36, 47]. This phenomenon is derived from the enhancement of Umklapp scattering processes which play a critical role in heat transport [8, 36, 47]. Additionally, when the temperature varies from 300 to 500 K, the *κ*_OPG-LZ_, *κ*_OPG-LA_, *κ*_OPG-ZZ_, and *κ*_OPG-ZA_ drops by 42%, 40%, 36%, and 37%, respectively. The dependence of TC of these two carbon allotropes on temperature shows that it is necessary to consider the temp effects for their practical applications.

The thermal properties of the two-dimensional materials, e.g., graphene [48, 49], silicene [34, 50, 51], and phosphorene [37], are sensitive to strain engineering. It has been reported that the TC of graphene with small size decreases with increasing tensile strain [48], and TC also can be enhanced by increasing strain when the sample is larger than 500 μm [49]. The unusual dependences of TC on sample size and strain is attributed to the competition between the boundary scattering and phonon-phonon scattering. In addition, the TC of silicene is found to increase at small tensile strain but decrease at large strain due to the competition between the phonon softening in the in-plane modes and phonon stiffening in the out-of-plane modes [34, 50, 51]. Therefore, it is significant and necessary to investigate the relationships between TC behavior and tensile strain for both OPG-L and OPG-Z structures.

We first investigate the mechanical properties of these two carbon allotropes. The sample size is about 5 nm long and 5 nm wide. To avoid any spurious high bond forces and nonphysical strain hardening [52, 53], the cut-off distance is fixed at (*R* = *S* = 1.95 Å). This cut-off distance in the modified optimized Tersoff potential is also consistent with that in previous Tersoff potentials (1.8–2.1 Å) [28, 53–55] that are being used to simulate C-C bond. All the simulations are initiated by relaxing atomistic configuration of structure to a minimum potential energy state. Uniaxial tensile strain is applied with the strain rate of 0.0002 ps^−1^. It should be noted that the interlayer equilibrium spacing of graphene (3.4 Å) is used to represent the interlayer equilibrium distance of the two structures. The mechanical properties of these two carbon allotropes are listed in Table 2, with comparison of graphyne and graphene [56]. The superscript characteristics of *z* and *a* represent zigzag and armchair sheets, respectively.

**Table 2:** Mechanical properties of carbon allotropes

Properties	OPG-L	OPG-Z	Graphyne [56]	Graphene [56]	Unit
Young’s modulus	538 ± 1.7^z^	492 ± 1.5^z^	503.1 ± 0.9^z^	856.4 ± 0.7^z^	Gpa
	648 ± 2.6^a^	550 ± 2.08^a^	525.0 ± 0.6^a^	964.0 ± 0.6^a^	Gpa
Ultimate strain (tension)	17.2%^z^	10.9%^z^	24.7%^z^	18.3%^z^	
	8.7%^a^	7.9%^a^	18.0%^a^	12.4%^a^	
Ultimate stress (tension)	84.61^z^	55.71^z^	89.02^z^	126.69^z^	Gpa
	48.99^a^	41.50^a^	68.79^a^	104.4^a^	Gpa

**Fig. 9:**
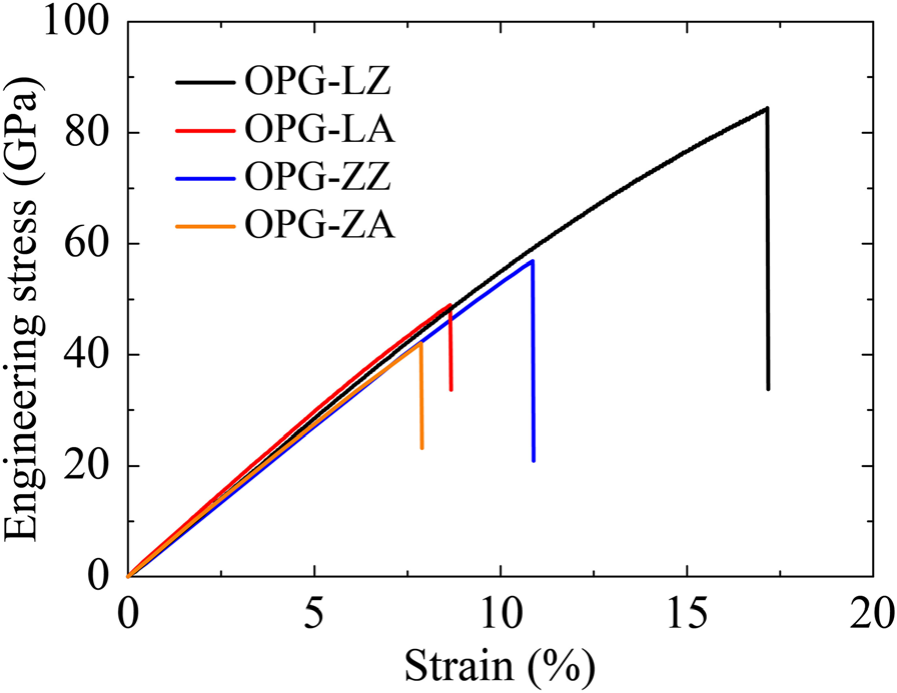
Stress-strain relationships of the two carbon allotropes along the zigzag and the armchair directions

It is seen from Table 2 that along the zigzag direction, the Young’s modulus of the OPG-L and OPG-Z are 538 GPa and 492 GPa, and along the armchair direction, the Young’s modulus are 648 GPa and 550 GPa, respectively. It indicates that the Young’s modulus of the OPG-L and OPG-Z are close to that of graphyne (503.1^z^ and 525.0^a^) but lower than that of graphene (856.4^z^ and 964.0^a^). Stress-strain relationships of the two carbon allotropes along the zigzag and the armchair directions are shown in Fig. 9. According to the fracture behaviors of these two carbon allotropes, we further obtain the ultimate strain (tension) of these two carbon allotropes. Respectively, along the zigzag direction, the ultimate strain (tension) of the OPG-L and the OPG-Z are 17.2% and 10.9%, and along the armchair direction, the ultimate strain (tension) are 8.7% and 7.9%. We found that the structure of OPG-L has higher strength under tensile strain in the zigzag direction. However, compared with graphyne and graphene, the ultimate strains (tension) of the two carbon allotropes are lower.

**Fig. 10:**
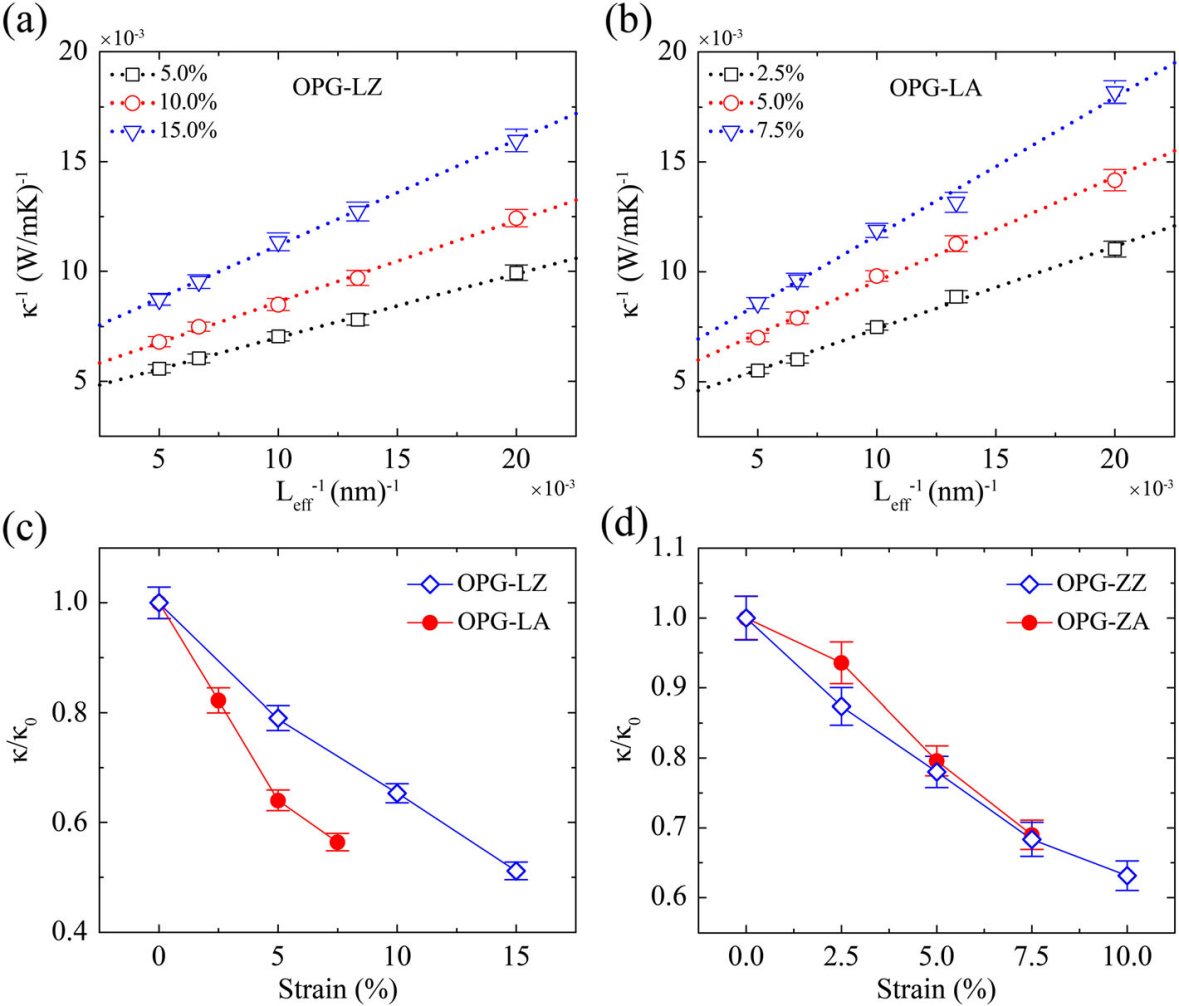
Inverse TC of **a** OPG-LZ, **b** OPG-LA at various uniaxial strains as a function of the inverse sample length, and the relative TC (*κ*/*κ*_0_) of **c** OPG-L and **d** OPG-Z as a function of strain. *κ*_0_ is the TC of stress free at 300 K, which is 310 W/mK, 332 W/mK, 247 W/mK, and 227 W/mK for *κ*_OPG-LZ_, *κ*_OPG-LA_, *κ*_OPG-ZZ_, and *κ*_OPG-ZA_, respectively

We then study the strain effect on TC of these two carbon allotropes by applying uniaxial tensile strain along the heat transfer direction. Simulation samples have the same width of 3 nm but different length of 50 nm, 75 nm, 100 nm, 150 nm, and 200 nm, respectively. The thermal conductivities of an infinite sample at various strains are extracted by doing extrapolation procedure (see Fig. 10a, b). As illustrated in Fig. 10c, d, all the converged thermal conductivities are normalized by the TC of stress free at 300 K (*κ*_0_), we further give the relative TC (*κ*/*κ*_0_) of the two carbon allotropes as a function of various uniaxial strains. Figure 10 clearly shows that the TC of both OPG-L and OPG-Z decreases monotonically with increasing tensile strain, which is consistent with previous studies in graphene [34, 48] but in sharp contrast to silicene [34, 50, 51] and phosphorene [37]. As shown in Fig. 10, the maximum reduction of *κ*_OPG-LZ_, *κ*_OPG-LA_, and *κ*_OPG-ZZ_, *κ*_OPG-ZA_ are 49%, 44%, 37%, and 31%, respectively. Particularly, the TC of OPG-L along the zigzag direction can be tuned through strain in a large range.

In order to further elucidate the strain effect on thermal transport properties of OPG-L and OPG-Z, we calculate the VDOS of phonons of OPG-LZ at typical strain. The VDOS are calculated by a Fourier transform of the autocorrelation function of atomic velocity. The function is defined as follows:

6 $$ P\left(\omega \right)=\frac{1}{\sqrt{2\pi }}\underset{0}{\overset{\infty }{\int }}{e}^{i\omega t}\left\langle \sum \limits_{j=1}^N{v}_j(t){v}_j(0)\right\rangle dt, $$

**Fig. 11:**
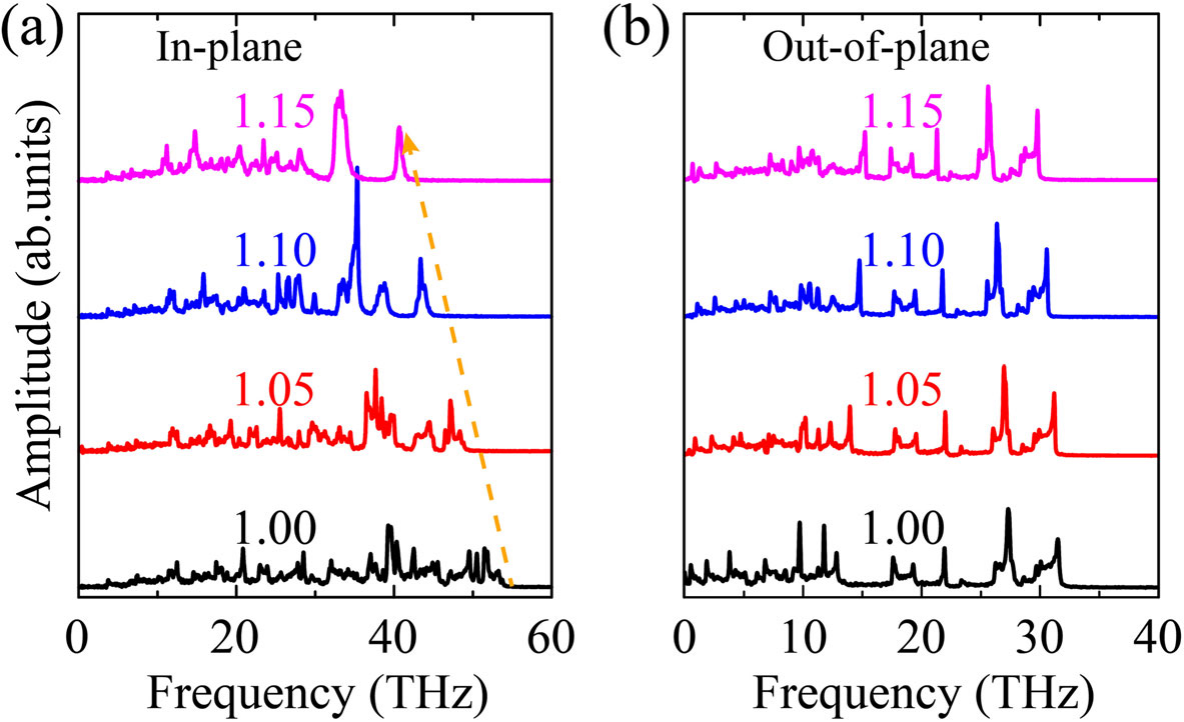
In-plane **a** and out-of-plane **b** VDOS of OPG-L versus uniaxial tensile strain along the zigzag direction

As illustrated in Fig. 11, the phonon softening (red shift) in in-plane and out-of-plane directions is observed. This phenomenon is in good agreement with previous studies in graphene under tensile strain [34, 48]. Particularly, compared with the VDOS in out-of-plane direction, the phonon softening in in-plane direction is obvious. It indicates that the decline of TC of OPG-L and OPG-Z is mainly owing to the strain-induced phonon softening in in-plane direction.

## Conclusions

In summary, both EMD and rNEMD simulations have been performed to investigate the thermal properties of the two novel 2D carbon allotropes composed of octagons and pentagons. The size, temperature, and strain effects on TC are obtained. Our results reveal that the TC increases monotonically with increasing size. The thermal conductivities of infinite sizes are obtained by linear relationships of the inverse length and inverse TC. The converged TC obtained by extrapolation in the reverse non-equilibrium molecular dynamics method is found to be in reasonable agreement with that in the equilibrium molecular dynamics method. The much lower TC, compared with graphene, is attributed to the lower phonon group velocity and phonon mean free path. Our findings provide important insights for the effects of size, temperature, and strain on thermal transport properties of OPG-L and OPG-Z, and indicate potential applications in thermal management devices in micro/nanoelectronics fields.

## Abbreviations

558: Five-five-eight-membered rings
OPG-L: Octagon and pentagon graphene-line
OPG-Z: Octagon and pentagon graphene-zigzag
rNEMD: Reverse non-equilibrium molecular dynamics
TC: Thermal conductivity
VDOS: Vibrational density of states
